# Serum Total Bilirubin Level Is Associated With Hospital Mortality Rate in Adult Critically Ill Patients: A Retrospective Study

**DOI:** 10.3389/fmed.2021.697027

**Published:** 2021-10-04

**Authors:** Zhou-Xin Yang, Xiao-Ling Lv, Jing Yan

**Affiliations:** Department of Critical Care Medicine, Zhejiang Hospital, Hangzhou, China

**Keywords:** critically ill patients, serum total bilirubin, mortality, logistic regression analysis, propensity score matching

## Abstract

**Background:** Serum bilirubin level has been suggested to be associated with mortality for patients with severe sepsis. This study aimed to investigate the association of serum total bilirubin level with hospital mortality rate in adult critically ill patients.

**Method:** Data were extracted from the Medical Information Mart for Intensive Care-III (MIMIC-III) database. Patients with measured serum total bilirubin levels that recorded within 24 h after admission were involved in this study. Association of serum total bilirubin level and hospital mortality rate was assessed using logistic regression analysis. Propensity score-matching (PSM) was used to minimize differences between different groups.

**Results:** A total of 12,035 critically ill patients were herein involved. In patients with serum total bilirubin level ≥ 2 mg/dL, the hospital mortality rate was 31.9% compared with 17.0% for patients with serum total bilirubin level < 2 mg/dL (546/1714 vs. 1750/10321, *P* < 0.001). The results of multivariable logistic regression analysis showed that the odds ratio of mortality in patients with serum total bilirubin level ≥ 2 mg/dL was 1.654 [95% confidence interval (CI): 1.307, 2.093, *P* < 0.001]. After propensity score matching, in patients with serum total bilirubin level ≥ 2 mg/dL, the weighted hospital mortality rate was 32.2% compared with 24.8% for patients with serum total bilirubin level < 2 mg/dL, *P* = 0.001).

**Conclusions:** Serum total bilirubin concentration was found to be independently associated with hospital mortality rate in adult critically ill patients.

## Introduction

In clinical treatment, it is highly essential to indicate the significance of early admission for critically ill patients. Several risk factors, e.g., obesity and high lactate level, were identified to be associated with the mortality of critically ill patients (1, 2).

Organ failure may result in death in intensive care unit (ICU), hence, the indicators of organ failure need to be highly taken into account for diagnosing critically ill patients. Serum creatinine and blood urea nitrogen (BUN) tests indicate kidney function, and can independently predict mortality of patients in ICU (3, 4). Troponin, which is associated with heart function, can predict the mortality of critically ill patients as well (5). It is therefore suggested that liver function may be associated with outcome of critically ill patients.

A patient's liver function can be indicated by bilirubin test. Bilirubin is a yellow compound that occurs in the normal catabolic pathway that breaks down heme in vertebrates. This catabolism is a necessary process in the body's clearance of waste products that arise from the destruction of aged or abnormal red blood cells. Total bilirubin was used in most of studies, which include direct bilirubin and indirect bilirubin. High bilirubin level may cause irreversible damage to brain and central nervous system (6, 7). However, bilirubin has been shown to be a potent antioxidant that is capable of potentially reversing or preventing damage due to reactive oxygen species (ROS) generated from ischemia and reperfusion (8). Bilirubin is highly important for clinical diagnosis of jaundice, as well as being an indicator of liver function. It has been reported that serum bilirubin test may indicate poor outcomes in patients with severe sepsis (9), traumatic brain injury (10), and COVID-19 patients (11). may also be helpful for diagnosis of acute respiratory distress syndrome (12).

To our knowledge, whether serum total bilirubin level could independently predict the clinical outcome of adult critically ill patients has remained elusive. In the present study, we aimed to investigate the association of serum total bilirubin level with the mortality and length of stay in hospital (particularly ICU) for adult critically ill patients.

## Methods

### Data Extraction

The Medical Information Mart for Intensive Care III (MIMIC III) is a large, freely-available database comprising de-identified health-related data associated with over forty thousand patients who stayed in critical care units of the Beth Israel Deaconess Medical Center (Boston, MA, USA) between 2001 and 2012 (13, 14). The database was approved by the Institutional Review Boards of Beth Israel Deaconess Medical Center (Boston, MA, USA) and the Massachusetts Institute of Technology (Cambridge, MA, USA). Informed consent was waived because patients in MIMIC-III database were de-identified for privacy protection. Data were extracted from the database by ZXY and XLL.

### Study Subjects and Clinical Outcomes

**Figure 1:**
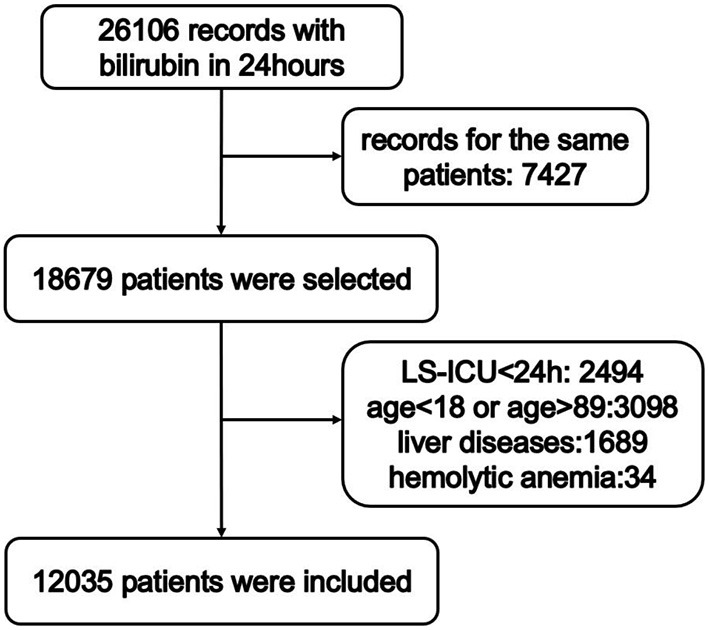
Overview of patients' selection. LS-ICU, length of stay in ICU.

The selection of study subjects is shown in Figure 1. Patients from either ICU type with measured serum total bilirubin levels that recorded within 24 h after admission were screened. For patients with more than one record, only the first-time admission was included. Patients with the following conditions were excluded: stayed in ICU for <24 h, younger than 18 or older than 89 years old, with liver diseases (e.g., chronic viral hepatitis B, chronic hepatitis C, esophageal varices, alcoholic fatty liver, autoimmune hepatitis, or liver cirrhosis), with hemolytic anemia. Diagnosis of liver diseases and hemolytic anemia was carried out according to 9th revision of the International Classification of Diseases code. Based on previous studies on bilirubin (15, 16), Patients were stratified into two cohorts according to maximum concentrations of total bilirubin 24 h after admission as follows: (1) serum total bilirubin level < 2 mg/dL, (2) serum total bilirubin level ≥ 2 mg/dL. The primary outcome in the present investigation was in-hospital mortality. Length of stay in hospital (LSH) and length of stay in ICU (LS-ICU) were defined as secondary outcomes. Continuous variables were replaced with their median values if <5% of the values were missing. For variables with more than 5% of values missing, the missing values were not imputed.

### Statistical Analysis

Evaluation of normality was performed using the histogram visual test. Continuous variables were described as median (25th, 75th percentile). Comparisons between two groups were carried out by the Mann-Whitney *U*-test. Categorical variables were presented as percentage (%) and compared by the chi-square test. A binary logistic regression analysis with forward likelihood-ratio test subset selection was used to search for independent factors associated with the mortality of critically ill patients. Variables used for regression included total bilirubin group, age, gender, body mass index (BMI), types of hospital admissions, diabetes mellitus, hypertension, renal failure, congestive heart failure, mean heart rate, mean blood pressure, mean respiratory rate, mean temperature, mean blood oxygen saturation (SpO2) level, maximum creatinine level, maximum glucose level, maximal lactate level, the highest level of BUN, and maximum number of white blood cells (WBC) recorded within 24 h. These variables were checked for multi-collinearity using variation inflation factor (VIF). Propensity score-matching (PSM) was used to minimize differences between the groups. A 1-to-6 nearest neighbor matching algorithm without replacement was applied in PSM, and caliper of width 0.1 was used for the matching. Age, gender, types of hospital admissions, diabetes mellitus, hypertension, renal failure, congestive heart failure, mean heart rate, mean blood pressure, mean respiratory rate, mean temperature, mean SpO2 level, maximum creatinine level, maximum glucose level, maximal lactate level, the highest level of BUN, and maximum number of WBC recorded within 24 h were selected to generate the propensity score with logistic regression. All matched patients with bilirubin level ≥ 2 mg/dL were assigned weight 1. When n patients with bilirubin level < 2 mg/dL were matched to one patient with bilirubin level ≥ 2 mg/dL, the patients with bilirubin level < 2 mg/dL were assigned weights 1/n. Balance checking after PSM were based on absolute standardized differences. Weighted conditional logistic regression analysis was used for the data after PSM using variables hospital mortality. LSH and LS-ICU were compared by the Wilcoxon signed rank test for the data after PSM, where, for patients with bilirubin level < 2 mg/dL in each matched set, averages were considered. Statistical analyses were undertaken using SPSS 22.0 (IBM, Armonk, NY, USA) and R3.6.2 Software. *P* < 0.05 was considered statistically significant.

## Results

### Patients' Baseline Characteristics

Among all admission records extracted from MIMIC-III database, 26,106 records were extracted. In addition, 7,427 cases were excluded due to they were not the first hospitalization. Furthermore, 6,644 patients were excluded because of the following reasons: LS-ICU shorter than 24 h after admission (2,494 records), patients who were younger than 18 years or older than 89 years old (3,098 records), patients with liver disease (1,689 records) and patients with hemolytic anemia (34 records). A total of 12,035 patients met inclusion criteria (Figure 1). There were 10,321 patients with serum total bilirubin level < 2 mg/dL, and 1,714 patients with serum total bilirubin level ≥ 2 mg/dL. Table 1 presents patients' clinical characteristic. Continuous variables were compared by the Mann-Whitney *U*-test and categorical variables were compared by the chi-square test. Patients with higher serum total bilirubin level were younger, and the percentage of males was higher. Besides, they have lower rates of hypertension and diabetes mellitus. Patients with higher serum total bilirubin level had higher heartrate, respiratory rate, levels of creatinine, lactate, and BUN, as well as the number of WBC, while had lower levels of blood pressure, temperature, SpO2, and glucose.

**Table 1:** Baseline characteristics of critically ill patients involved in the study.

**Variables**	**Total bilirubin < 2 mg/dL** **(*N* = 10,321)**	**Total bilirubin≥2 mg/dL** **(*N* = 1,714)**	***P*-value**
Total bilirubin (mg/dL)	0.600 (0.400, 0.900)	3.900 (2.600, 7.100)	
Age (year)	64.983 (52.071, 76.817)	62.013 (49.228, 75.511)	<0.001
**Gender**			
Female	4,501 (43.6%)	675 (39.4%)	0.001
Male	5,820 (56.4%)	1,039 (60.6%)	
BMI (kg/m2)	27.248 (23.744, 31.752)	27.267 (23.803, 31.412)	0.890
**Admission type**			
EMERGENCY	9,390 (91.0%)	1,499 (87.5%)	<0.001
URGENT	259 (2.5%)	70 (4.1%)	
ELECTIVE	672 (6.5%)	145 (8.5%)	
**Comorbidities**			
Diabetes	2,676 (25.9%)	368 (21.5%)	<0.001
Hypertension	1,205 (11.7%)	161 (9.4%)	0.006
Renal failure	1,443 (14.0%)	218 (12.7%)	0.161
Congestive heart failure	1,684 (16.3%)	307 (17.9%)	0.100
Mean heart rate (bpm)	86.089 (74.821, 98.146)	91.613 (79.542, 104.900)	<0.001
Mean blood pressure (mmHg)	78.038 (71.163, 86.304)	75.643 (69.226, 83.045)	<0.001
Mean respiratory rate(insp/min)	18.600 (16.375, 21.636)	19.884 (17.243, 23.262)	<0.001
Mean temperature (°C)	36.880 (36.500, 37.317)	36.822 (36.405, 37.315)	0.003
Mean Spo2 (%)	97.556 (96.149, 98.736)	97.074 (95.704, 98.400)	<0.001
Maximum Creatinine (mg/dl)	1.100 (0.800, 1.700)	1.300 (0.900, 2.300)	<0.001
Maximum Glucose (mg/dL)	158.000 (125.000, 211.000)	150.000 (119.000, 199.000)	<0.001
Maximum Lactate (mmol/L)	2.200 (1.500, 3.800)	3.100 (1.800, 5.700)	<0.001
Maximum BUN (mg/dL)	22.000 (15.000, 37.000)	28.000 (17.000, 48.000)	<0.001
Maximum WBC(109/L)	12.800 (9.200, 17.600)	13.300 (8.800, 19.800)	0.032

### Association of Serum Total Bilirubin Level With Clinical Outcomes

The patients' outcomes with serum total bilirubin levels ≥ 2 mg/dL or < 2 mg/dL are shown in Table 2. LSH and LS-ICU were compared by the Mann-Whitney *U*-test and in-hospital mortality was compared by the chi-square test. In patients with serum total bilirubin level ≥ 2 mg/dL, the hospital mortality rate was 31.9% compared with 17.0% for patients with serum total bilirubin level < 2 mg/dL (546/1714 vs. 1750/10321, *P* < 0.001). Besides, patients with higher serum total bilirubin level had a longer LS-ICU [3.240, interquartile range (IQR): 1.928, 7.170 vs. 2.865, IQR: 1.780, 5.586, *P* < 0.001] and LSH (9.635, IQR: 5.157; 17.560 vs. 7.209, IQR: 4.184, 12.989, *P* < 0.001).

**Table 2:** Outcomes of critically ill patients before and after propensity score matching.

**Variables**	**Before propensity score matching**			**After propensity score matching**		
	**Total bilirubin <2 mg/dL** **(*N* = 10321)**	**Total bilirubin≥2 mg/dL** **(*N* = 1,714)**	***P*-value**	**Total bilirubin < 2 mg/dL** **(*N* = 3,884)**	**Total bilirubin≥2 mg/dL** **(*N* = 901)**	***P*-value**
Hospital mortality	1,750 (17.0%)	546 (31.9%)	<0.001	789 (20.3%)	290 (32.2%)	0.001
LS-ICU (day)	2.865 (1.780, 5.586)	3.240 (1.928, 7.170)	<0.001	3.316 (1.953, 7.243)	3.702 (2.036, 8.495)	0.032
LSH (day)	7.209 (4.184, 12.989)	9.635 (5.157, 17.560)	<0.001	7.948 (4.505, 14.081)	9.158 (5.171, 16.708)	0.335

*Unweighted counts and percentages were presented for hospital mortality of patients after PSM. For patients before PSM, LSH and LS-ICU were compared by the Mann-Whitney U-test and in-hospital mortality was compared by the chi-square test. For patients after PSM, averages LSH and LS-ICU of patients with bilirubin < 2 mg/dL in each matched set were calculate and were compared with patients with bilirubin < 2 mg/dL by the Wilcoxon signed rank test. In-hospital mortality were compared by weighted conditional logistic regression with mortality as the outcome for patients after PSM*.

Logistic regression analysis was used to explore the association between serum total bilirubin level and hospital mortality rate (Table 3). The result of the multivariable logistic regression model with hospital mortality as the outcome was shown. The independent variables in the model were chosen by the forward selection. The odds ratio of mortality in patients with serum total bilirubin level ≥ 2 mg/dL was 1.654 (95% CI: 1.307, 2.093, *P* < 0.001). Other variables, such as the patients' age, heart rate, respiratory rate, mean temperature, as well as levels of lactate and BUN also showed high predicted value for hospital mortality (*P* < 0.001). The multi-collinearity was checked, and no obvious collinearity was noted.

**Table 3:** Predictors of mortality in critically ill patients.

**Variables**	**OR (95% CI)**	***P*-value**	**Collinearity (VIF)**
Total bilirubin group	1.654 (1.307, 2.093)	<0.001	1.082
Age	1.024 (1.018, 1.031)	<0.001	1.321
BMI (per 10 kg/m2)	0.883(0.781, 0.999)	0.048	1.089
Admission type			1.044
EMERGENCY	–	–	
URGENT	1.123 (0.653, 1.933)	0.674	
ELECTIVE	0.369 (0.243, 0.560)	<0.001	
Mean heart rate (bpm)	1.016 (1.010, 1.022)	<0.001	1.364
Mean blood pressure (mmHg)	0.985 (0.976, 0.995)	0.003	1.112
Mean respiratory rate (insp/min)	1.058 (1.035, 1.082)	<0.001	1.258
Mean temperature (°C)	0.751 (0.662, 0.853)	<0.001	1.201
Mean Spo2 (%)	0.952 (0.918, 0.988)	0.009	1.127
Maximum Lactate (mmol/L)	1.183 (1.149, 1.217)	<0.001	1.299
Maximum BUN (per 10 mg/dL)	1.108(1.071, 1.146)	<0.001	2.257

*OR, odds ratio; 95% CI, 95% confidence interval; VIF, variation inflation factor*.

### Outcomes After PSM

As the baseline levels were different between patients with serum total bilirubin levels ≥ 2 mg/dL and < 2 mg/dL, we used PSM to reduce these differences. After removing patients with missing values, 6,133 patients were used in PSM, of which 5,212 patients had bilirubin level < 2 mg/dL, and 921 patients had bilirubin level ≥ 2 mg/dL. For this purpose, 901 patients with total bilirubin ≥ 2 mg/dL and 3,884 patients with total bilirubin < 2 mg/dL were well-matched. Unweighted counts and percentages after PSM were presented for categorical variables in Table 4. The matching was appropriate, since absolute standardized differences were lower than 10% for all the 18 variables used for PSM. Unweighted counts and percentages were presented for in-hospital mortality of patients after PSM in Table 2. After PSM, the weighted hospital mortality rate was 32.2% in patients with serum total bilirubin level ≥ 2 mg/dL, compared with 24.8% for patients with serum total bilirubin level < 2 mg/dL. Conditional logistic regression analysis showed the differences between these two groups were significant (*P* = 0.001). LSH and LS-ICU were compared by the Wilcoxon signed rank test. Patients with higher serum total bilirubin level had a longer LS-ICU (3.702, IQR: 2.036, 8.495 vs. 3.316, IQR: 1.953, 7.243, *P* < 0.032). However, there was no significant difference in LSH between the two groups (*P* = 0.335) (Table 2).

**Table 4:** Balance checking for the variables after propensity score matching.

**Variables**	**Total bilirubin <2 mg/dL (*N* = 3,884)**	**Total bilirubin≥2 mg/dL (*N* = 901)**	**Weighted mean differences**	**Weighted** **absolute standardized differences (%)**
Age (year)	63.064 (47.452, 75.888)	61.025 (48.357, 74.906)	−0.200	1.1
Gender (Male)	2,247 (57.9%)	517 (57.4%)	−0.005	0.9
**Admission type**				
EMERGENCY	3,387 (87.2%)	790 (87.7%)	0.005	1.5
URGENT	152 (3.9%)	33 (3.7%)	−0.003	1.3
ELECTIVE	345 (8.9%)	78 (8.7%)	−0.002	0.8
**Comorbidities**				
Diabetes	796 (20.5%)	188 (20.9%)	0.004	0.9
Hypertension	303 (7.8%)	72 (8.0%)	0.002	0.7
Renal failure	407 (10.5%)	95 (10.5%)	0.001	0.2
Congestive heart failure	712 (18.3%)	162 (18.0%)	−0.003	0.9
Mean heart rate (bpm)	94.688 (82.487, 106.930)	94.152 (81.469, 107.084)	−0.364	2.0
Mean blood pressure (mmHg)	75.317 (68.980, 83.205)	75.290 (69.525, 82.220)	0.140	1.3
Mean respiratory rate (insp/min)	20.111 (17.320, 23.944)	20.138 (17.441, 23.829)	−0.034	0.7
Mean temperature (°C)	36.889 (36.426, 37.417)	36.865 (36.400, 37.356)	−0.002	0.2
Mean Spo2 (%)	97.330 (95.813, 98.636)	97.185 (95.770, 98.480)	0.091	2.2
Maximum Creatinine (mg/dl)	1.300 (0.900, 2.200)	1.300 (0.900, 2.450)	−0.021	1.1
Maximum Glucose (mg/dL)	161.000 (129.000, 212.000)	157.000 (123.000, 212.500)	−2.498	2.3
Maximum Lactate (mmol/L)	2.800 (1.637, 5.400)	3.000 (1.800, 5.500)	0.052	1.4
Maximum BUN (mg/dL)	25.000 (16.000, 47.000)	28.000 (17.000, 48.500)	−0.434	1.5
WBC (10^9^/L)	14.300 (9.800, 20.100)	13.900 (9.400, 20.250)	−0.025	0.3

*Unweighted counts and percentages were presented for categorical variables of patients after PSM. Weighted mean differences and weighted absolute standardized differences between two groups were shown*.

## Discussion

Seeking for indicators for critically ill patients' outcome is of great clinical significance. In the current study, we found that bilirubin could independently predict high mortality. In contrast to previous researches that concentrated on patients with severe sepsis, critically ill patients suffering from several diseases were included in the present study. Therefore, our results could possibly be used to predict the death risk in the majority of patients in ICU.

The results of the present study revealed that elevated serum total bilirubin level results in high rate of hospital mortality. In addition to consideration of elevated bilirubin as an indicator of liver damage (17) or disease or certain types of anemia (18), the bilirubin toxicity could also be a main cause of death of critically ill patients. Several differences were found (e.g., comorbidities of diabetes/hypertension, heartrate, and levels of creatinine and lactate) between patients with high level of bilirubin and those with low level of bilirubin before PSM. These differences might be related to heterogeneity of the patient population. However, the differences might be associated with different levels of bilirubin in study subjects.

Numerous methods were presented to reduce the serum levels of bilirubin (19, 20). However, reduction of bilirubin level is not always beneficial for patients. Low level of bilirubin might be associated with ulcerative colitis, probably due to role of bilirubin in removing ROS (21). Although a number of scholars reported selective bilirubin removal for patients with jaundice-related kidney injury (22) and those who require to undergo cardiac surgery (23), further studies should be performed to indicate whether the reduction of serum total bilirubin level is advantageous for patients in ICU.

Although the present study showed that serum total bilirubin level might be used to predict the mortality of the critically ill patients, scoring methods [e.g., sequential organ failure assessment (SOFA)] could be more reasonable for diagnosis of such patients. SOFA score is used to track a person's status during the stay in an ICU to determine the extent of a person's organ function or rate of failure, while serum total bilirubin level only shows the function of the liver. Nevertheless, the independent predictive ability of scoring systems for mortality is relatively complex.

There are several limitations in the current study. Firstly, records extracted from MIMIC database were de-identified, and patients' clinical records were limited, especially patients who aged over 89 years old, thus, our study did not include such patients' clinical records. Secondly, we excluded patients with liver disease and hemolytic anemia, therefore, our results cannot be applicable to those patients. Thirdly, although PSM was used to support our results, it could not overcome the limitation of the retrospective nature of this study.

In conclusion, we showed that the serum total bilirubin level could predict hospital mortality rate for critically ill patients. However, further prospective researches are required to clarify the mechanism underlying the prediction of mortality of critically ill patients by serum total bilirubin level.

## Data Availability Statement

Publicly available datasets were analyzed in this study. This data can be found here: https://mimic.physionet.org/.

## Ethics Statement

Ethical review and approval was not required for the study on human participants in accordance with the local legislation and institutional requirements. Written informed consent for participation was not required for this study in accordance with the national legislation and the institutional requirements.

## Author Contributions

Z-XY extracted the data and wrote the draft of the manuscript. JY designed the study and revised the manuscript. X-LL performed statistical analyses. All authors contributed to the article and approved the submitted version.

## Funding

This study was supported by the National Natural Science Foundation of China (Grant No. 81801902), Natural Science Foundation of Zhejiang Province (Grant No. LY21H150002).

## Conflict of Interest

The authors declare that the research was conducted in the absence of any commercial or financial relationships that could be construed as a potential conflict of interest.

## Publisher's Note

All claims expressed in this article are solely those of the authors and do not necessarily represent those of their affiliated organizations, or those of the publisher, the editors and the reviewers. Any product that may be evaluated in this article, or claim that may be made by its manufacturer, is not guaranteed or endorsed by the publisher.
